# The Predictive Role of Perceived Autonomy Support in Elementary School Children Physical Activity

**DOI:** 10.3390/children9101592

**Published:** 2022-10-21

**Authors:** Mikel Vaquero-Solís, Miguel Ángel Tapia-Serrano, Pedro Antonio Sánchez-Miguel, Rubén Llanos-Muñoz, Miguel Angel López-Gajardo

**Affiliations:** 1 Grupo Análisis Comportamental de la Actividad Física y el Deporte (ACAFYDE), Department of Didactics of Musical, Plastic and Corporal Expression, Teaching Training College, University of Extremadura, 10071 Cáceres, Spain; 2 Grupo Análisis Comportamental de la Actividad Física y el Deporte (ACAFYDE), Department of Didactics of Musical, Plastic and Corporal Expression, Faculty of Sports Sciences, University of Extremadura, 10003 Cáceres, Spain

**Keywords:** perceived autonomy support, autonomous motivation, physical activity, children, self-determination theory

## Abstract

The present article aimed to test a predictive model based on children’s perception of autonomy support exercised by their physical education teachers in establishing a state of high motivational quality, which in turn leads to greater intention and physical activity. Participants were 502 elementary school students aged 9 to 11 years (52.59% males (9.47 ± 0.53 years old) and 47.41% females (9.54 ± 0.53 years old)), who completed a self-reported questionnaire of perceived autonomy support from physical education teachers, self-determination, intention to be physically active, and physical activity. Results showed that perceived autonomy support was positively related to autonomous motivation, which in turn was associated with intention and physical activity. In addition, the model did not present variations with respect to gender and confirmed the indirect effects of autonomous motivation on intention and practice of physical activity. Finally, we conclude on the importance of an adequate perception of autonomy support in students by their teachers for the establishment of an appropriate motivational state that could promote greater intention and physical activity. Thus, the motivational state acquired in physical education classes can be translated into intention and practice of physical activity in different contexts of students’ daily lives.

## 1. Introduction

Several studies show that boys and girls do not get enough physical activity (PA) [1]. For this reason, important research suggests that insufficient PA is one of the causes associated with several physical and mental health problems (e.g., obesity, cardiovascular problems, diabetes, life satisfaction, stress, depression, etc.) [2,3]. Given the importance to promote PA among youth, different public health organizations, educators, and other actors in the school context have sought to identify and promote optimally effective strategies, such as supporting health-related experiences, to enhance PA in this population [4,5]. Therefore, physical education (PE) has been identified as an existing resource with great potential through which to promote PA, both in and out of school, in young people [6]. In this regard, the PE teacher has special relevance to motivate the students to perform and maintain healthy behaviours, such as PA. Previous research shows that the autonomy support provided by the teacher through different actions (i.e., considering students’ suggestions and interests, giving them the opportunity to choose, providing them with adequate justification for justified actions, etc.) can have important effects on the motivational climate, intention to be physically active, and PA [7,8]. These results are in agreement with the self-determination theory (SDT) [9], which postulates that satisfaction of basic needs, especially support and competence needs, predict a desired motivational state and other positive outcomes, such as well-being, engagement, and persistence.

Concerning SDT, [10] proposes how the behaviours are voluntary or self-determined according to the basic psychological needs (i.e., autonomy, competence, and relatedness) satisfaction or thwarting. Satisfaction of the need for autonomy refers to the desire to experience freedom or volition in the choice of one’s actions. The need for competence makes reference to the desire to feel effective in interacting with the environment through one´s own capacities. Finally, relatedness indicates being securely attached and meaningfully connected with individuals or groups [11]. Therefore, the higher the degree of satisfaction that students possess of these needs, the higher their level of motivation along the continuum of self-determination [12]. In this regard, Ryan et al. [13] pointed out that motivational behaviour can be divided into three stages: Controlled motivation, autonomous motivation, and amotivation. Likewise, the different types of motivational stages are determined by different forms of regulation in what is known as the self-determination continuum. For controlled motivation, behaviours are determined by introjected regulation (performing an action to avoid feelings of guilt or anxiety) and external regulation (performing an activity to obtain a reward or avoid punishment). In autonomous motivation, behaviours will be determined by intrinsic regulation (the performance of an activity for mere enjoyment or because it is a challenge), identified regulation (performing an activity that is aligned with the person’s values), and integrated regulation (when the activity is totally assimilated with the individual’s meaning). Finally, amotivation is presented, which reflects the absence of motivation.

In the context of PA, the systematic review and meta-analysis conducted by Vasconcellos et al. [14] showed that autonomy support provided by the teacher influences autonomous motivation generated in students. In this sense, both cross-sectional studies and quasi-experimental studies have shown how autonomy support affects motivational quality [15,16,17,18]. Similarly, Vasconcellos et al. [14] evidenced that controlled motivation, through introjected regulation, was positively related to autonomy support. In the same line, the works completed by Haerens et al. [12] and Leo et al. [19] also confirmed these relationships, wherein perceived autonomy support was positively and significantly related to controlled motivation.

Regarding the role of autonomous motivation in relation to adaptive behaviours, such as intention or PA [14], numerous studies confirm the positive associations between more autonomous types of regulation and PA intention and PA [15,17,18,20]. Specifically, the work conducted by Schneider et al. [18] showed how the motivational quality generated through autonomy support has an indirect effect on the intention to be physically active and PA in a sample of adolescents. In the same way, the work conducted by Leo et al. [19] confirmed the predictive value of motivational quality on the intention to be physically active. This fact gives strength to a theoretical model, wherein intention predicts PA [18,21,22]. In this sense, several studies about autonomy support perception confirm the mediator value of intention on PA [15,18,20]. In addition, other studies have shown the importance of autonomy support on PA, cognitive components, and different aspects of well-being (e.g., quality of life, affect, healthy habits, etc.) [8,17,21].

In spite of the findings on the role of autonomy support in various aspects of PA and well-being, no work has tested these relationships in a school population of children, as was suggested in the research by Vasconcellos et al. [14]. Thus, the present work aims to test a model in which students’ perceptions of the autonomy support they receive from the teacher are related to higher motivational quality, which leads to greater intention on PA and PA in the future. Taking into account these purposes, the following hypotheses were tested: First, it was hypothesized (H1) that children’s perceived autonomy support will be positively related to autonomous motivation. Second, it was hypothesized (H2) that autonomous motivation perceived by children will be positively associated with intention to be physically active. Third, it was expected (H3) that intention to be physically active will be associated with PA in children. Fourth, it was expected (H4) that autonomous motivation will be a mediator in the relationship between children’s perceived autonomy support and intention to be physically active (H4a) and PA (H4b). In addition, due to differences in previous studies [12,19], in a more exploratory fashion, this paper aims to analyse whether there are gender differences in the hypothesized model.

## 2. Material and Method

### 2.1. Design and Participants

A cross-sectional design was carried out in four primary schools in a city in south-western Spain. Schools were selected by purposive sampling based on the proximity of the researchers’ work centre to the schools. The baseline data were collected from March 2022 to April 2022. A convenience sample of 502 school children, aged to 9 to 11 years (9.50 ± 0.53 years old), in their fourth and fifth year of primary education, participated in this study. Of the total sample, 264 were boys (52.59%; 9.47 ± 0.53 years old), and 238 were girls (47.41%; 9.54 ± 0.53 years old). In addition, note that the sample size was calculated through the following formula: *n* = *n* = (Z)2(*p* (1 − *p*)e2), where “*n*” is the sample size, Z = 1.96 (95% confidence interval), *p* = number of 4th- and 5th-grade primary school students in the city where the study was conducted (3344 students), and e = margin of error (3%). The minimum sample size (considering a 10% non-response) was 345 school children. Finally, the inclusion criteria used for sample selection were as follows: (i) Primary school was located in the region of Extremadura, specifically in Cáceres; (ii) permission was granted by the director and the head of studies of physical education to participate in the project; (iii) students were enrolled in the 4th and 5th grades of primary education; (iv) consent was given by the parents and the students to participate; (v) the students were not participating in a previous program of similar characteristics during the intervention.

### 2.2. Measures

***Sociodemographic characteristics.*** Age (in years), sex (male or female), and socioeconomic status (SES) were self-reported. The Family Affluence Scale-II (FAS II) [23] was used to assess children’s SES. In addition, its score was obtained on a range from 0 to 9 based on responses to four questions.

***Perceived autonomy support for physical activity.*** Students’ perceptions of autonomy support for PA from their PE teacher were assessed by the Spanish version [24] of the Perceived Autonomy Support Scale for Exercise Settings (PASSES) [25]. This scale consisted of twelve items intended to measure the student’s perception of the PE teacher’s autonomy support for PA (e.g., I feel that the physical education teacher gives me choices and opportunities about whether to do sports and/or vigorous exercise in my free time). The responses corresponded to a Likert-type scale ranging from 1 (strongly disagree) to 7 (strongly agree). Support for the PE teacher’s autonomy was measured as the mean of all responses. A high mean score indicates that the student perceives a higher perception of autonomy support towards PA from his/her PE teacher. A confirmatory factor analysis (CFA) was conducted to check validity, showing adequate model fit: χ^2^ = 116.997, df = 54, *p* < 0.001, CFI = 0.96, TLI = 0.95, RMSEA = 0.05, 95% CI (0.036, 0.060), SRMR = 0.03. In addition, internal consistency values were adequate for a single first-order factor (α = 0.92, ω = 0.92).

***Motivation in physical activity.*** Students’ perceptions of different behavioural regulations were assessed by Behavioral Regulation Exercise Scale (BREQ-3) [26], adapted to the Spanish PA context. The instrument is preceded by the stem “I participate in PA…” and followed 23 items (four items per factor) that measure intrinsic motivation (e.g., because I think exercise is fun), integrated (e.g., because it agrees with my way of life), identified (e.g., because I value the benefits that physical exercise has), introjected (e.g., because I feel guilty when I do not practice it), external regulation (e.g., because others tell me I should do it), and amotivation (e.g., I do not see why I have to do it). Responses correspond to a Likert-type scale ranging from 0 (not very true) to 4 (very true). The hierarchical confirmatory factor analysis (HCFA) of the data offered support for this three-factor structure showing acceptable model fit: χ^2^ = 392.390, df = 156, *p* < 0.001, CFI = 0.91, TLI = 0.90, RMSEA = 0.05, 95% CI (0.048, 0.062), SRMR = 0.08. Furthermore, internal consistency values were deemed acceptable: α = 0.81 and ω = 0.81 for autonomous motivation, α = 0.83 and ω = 0.84 for controlled motivation, and α = 0.80 and ω = 0.80 for amotivation.

***Intention to be physically active.*** Intention to be physically active was measured using the Measurement of the Intention to be Physically Active (MIFA) for Spanish children [27]. This scale is an adaptation of the Intention to be Physically Active Scale (IPAS) [28]. This questionnaire was composed of five items aimed at measuring the individual’s intention to be physically active after school (e.g., I am interested in developing my physical fitness). The items were preceded by the following sentence: “Regarding your intention of doing PA”. Responses correspond to a Likert-type scale ranging from 1 (strongly disagree) to 5 (strongly agree). The CFA of the data offered support for this factor structure showing acceptable model fit: χ^2^ = 4.444, df = 5, *p* = 0.487, CFI = 1.00, TLI = 1.00, RMSEA = 0.00, 95% CI (0.000, 0.058), SRMR = 0.02. The final score was the mean of its items. Furthermore, internal consistency values were adequate for a single first-order factor (α = 0.72, ω = 0.72).

***Physical activity.*** PA was assessed using the Spanish version of the PA Questionnaire for Children (PAQ-C) [29]. This questionnaire has been shown to be valid (r = 0.30–0.40) [30] and reliable (α = 0.76 and intraclass correlation coefficient (ICC) = 0.96) for assessing PA in Spanish children [31]. In this study, Cronbach’s alpha for PA on this scale was 0.88. This questionnaire is composed of nine questions, which evaluate PA performed during the last 7 days. Each answer is scored on a 5-point scale ranging from 1 to 5. The PA score was measured as the average value of all responses. Higher scores indicate higher levels of PA.

### 2.3. Procedure

This study was conducted in accordance with the ethical principles of the Declaration of Helsinki and the Ethics Committee of the lead author’s University (120/2018). In this sense, all participants were treated in accordance with the ethical guidelines of the American Psychological Association, respecting their freedom of participation, confidentiality, and anonymity of responses. Regarding the procedure carried out for data collection, the research team contacted the principals and physical education teachers at each school to conduct the study. Subsequently, after acceptance of participation by the principals and teachers, a letter of consent was designed for the parents or legal guardians of the students, who had to return it, signed, to authorize their collaboration in the study. Once the permits and informed consent were obtained, data were taken. In addition, we developed several procedures to the extent of common method bias, attributing the results to the hypothesized model between variables [32]. For example, we reminded the respondents that their answers were anonymous, and participation in the survey was voluntary. In addition, we used only standardized scales and different scale end points [32].

The participants filled out the questionnaires during a PE class, individually and in a suitable climate that allowed them to concentrate without having any type of distraction. A member of the research team was present to provide help and answer to any of the questions that students might have. The time to complete the survey was approximately 45 min.

### 2.4. Data Analyses

Two different statistical packages, IBM SPSS V.23 (New York, NY, USA) and Mplus version 7.3 (Los Angeles, CA, USA) [33], were used to perform the statistical analyses. First, a CFA was performed for each scale to show the acceptable fit of all the variables that would compose the model. Subsequently, descriptive statistics and bivariate correlations were performed. For the main analyses, structural equation modelling (SEM) was used to test the hypothesized and alternative models (i.e., the relations between perceived autonomy support, motivation, intention to be physically active, and PA). Significance was set at *p* < 0.05 for all analyses. The robust maximum likelihood (MLR) estimator was used, as it is robust for non-normal observations and can handle random missing data [34]. In MacKinnon, Lockwood, and Williams [35], direct effects were tested using the bias-corrected bootstrap method (10,000 samples with 95% bias-corrected confidence intervals (CIs); with the maximum likelihood procedure (MLR); bootstrapping is unavailable using MLR estimation). This currently represents the most effective way to identify mediated relations, given the asymmetry of their theoretical distributions [36]. The mediated relation is considered significantly different from zero when the CI does not cross zero.

## 3. Results

### 3.1. Descriptive Statistics

Means, standard deviations, reliability analysis, intraclass correlation coefficients (ICC), and correlations of the variables included in the investigation are presented in Table 1. Measures showed acceptable levels of reliability (α and ω > 0.70; Nunnally and Bernstein, 1994). In addition, the ICCs—which show the degree of shared variance due to classroom membership—were very low for almost all variables (ICCs = 0.02–0.20; see Table 1). As for the bivariate correlations, participants reported a significant and positive association between all variables (*p* < 0.01), except the significant and negative relationship between amotivation and intention to be physically active (*p* < 0.05). In addition, the perceived autonomy support and autonomous motivation did not show a significant association with amotivation (*p* > 0.05).

### 3.2. Main Analysis

**Figure 1:**
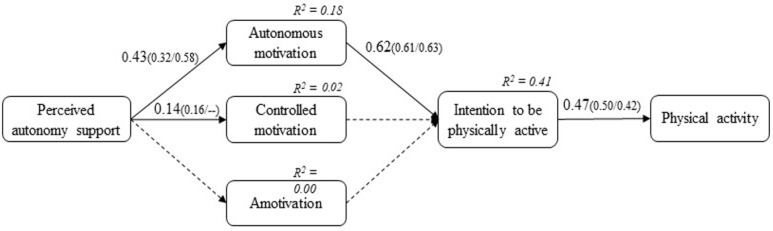
SEM of the hypothesized model for the full sample and for boys (first coefficient in brackets) and girls (second coefficient in brackets). Note: The coefficients shown are normalized and statistically significant at the 0.05 level. For reasons of parsimony, relationships that were not statistically significant are not shown. The relationship between autonomous motivation and controlled motivation (r = 0.24, *p* < 0.001; *r* = 0.22, *p* < 0.001 for boys, and *r* = 0.24, *p* < 0.001 for girls), autonomous motivation and amotivation (*r* = 0.01, *p* = 0.947; r = 0.01, *p* = 0.934, and *r* = 0.04, *p* = 0.834 for girls), and the relationship between controlled motivation and amotivation (*r* = 0.77, *p* < 0.001; *r* = 0.77, *p* < 0.001 for boys, and *r* = 0.78, *p* < 0.001 for girls). Non-significant paths were indicated with dashed lines.

SEM was used to test the different relationships among the variables represented in the model (see Figure 1). Firstly, the model showed an adequate fit to the data: χ^2^ (653) = 1280.477, *p* < 0.001, CFI = 0.90, TLI = 0.90, RMSEA = 0.04 (95% CI (0.04, 0.05)), SRMR = 0.08. Secondly, the results showed that perceived autonomy support positively predicted autonomous (*β* = 0.43; *p* < 0.001; 95% CI (0.33–0.53)) and controlled motivation (*β* = 0.14; *p* = 0.006; 95% CI (0.04–0.24)). Thirdly, only autonomous motivation positively predicted intention to be physically active (*β* = 0.62; *p* < 0.001; 95% CI (0.47–0.78)). Finally, intention to be physically active presented a positive effect with the PA (*β* = 0.47; *p* < 0.001; 95% CI (0.37–0.57)).

In addition, we included gender as a covariate in the same model (i.e., one model within males and females) to examine the relations in the hypothesized models. Figure 1 shows the model coefficients obtained for men and women (first and second coefficient in brackets, respectively). As can be noticed, all the paths (with few exceptions) were statistically significant across the two genders. A comparison of the two groups revealed that all the relationship were statistically significant across the two genders, except the positive relation between perceived autonomy support and controlled motivation that we found in the full sample, which was significant among males (*β* = 0.16; *p* = 0.027; 95% CI (0.02–0.30)) but not among females (*β* = 0.12; *p* = 0.071; 95% CI (−0.10–0.25)).

Regarding to the mediating effects of students’ motivation between perceived autonomy support and intention to be physically active and PA, a significant indirect effect was observed in the relationship of students’ autonomous motivation and intention to be physically active (*β*  = 0.26, *p* ≤ 0.001, 95% CI (0.17, 0.36); for boys → *β* = 0.19; *p* = 0.006; 95% CI (0.08–0.33) and for girls → *β* = 0.35; *p* < 0.001; 95% CI (0.11–0.53)) and PA in children (*β*  = 0.16, *p* ≤ 0.001, 95% CI (0.09, 0.24); for boys → *β* = 0.12; *p* = 0.006; 95% CI (0.05–0.20) and for girls → *β* = 0.22; *p* = 0.011; 95% CI (0.08–0.36)), respectively. However, students’-controlled motivation and amotivation did not show a significant mediating effect between perceived autonomy support and intention to be physically active (*β* = 0.00–−0.01, *p* = 0.970–0.845, 95% CI (−0.02, 0.03–−0.01, 0.05)) and PA, respectively (*β* = 0.00–0.01, *p* = 0.816–0.602, 95% CI (−0.04, 0.01–−0.03, 0.02)).

**Figure 2:**
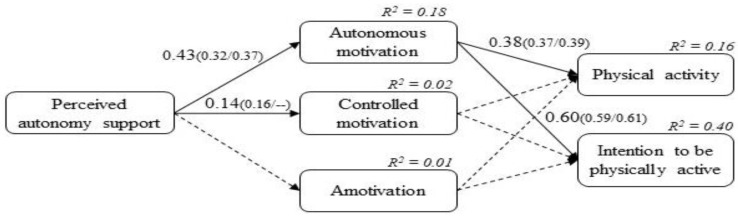
SEM of the alternative model for the full sample and for boys (first coefficient in brackets) and girls (second coefficient in brackets). Note: Coefficients are standardized and statistically significant at 0.05 level. As in Figure 1, relationships that were not statistically significant were not shown for reasons of parsimony. Following, R coefficients are shown for the relationship between autonomous motivation and controlled motivation (r = 0.24, *p* < 0.001; r = 0.22, *p* < 0.001 for boys, and r = 0.24, *p* = 0.001 for girls), between autonomous motivation and amotivation (r = 0.01, *p* = 0.935; r = 0.01, *p* = 0.936, and r = 0.04, *p* = 0.879 for girls), and the relationship between controlled motivation and amotivation (r = 0.77, *p* < 0.001; r = 0.77 *p* < 0.001 for boys, and r = 0.78, *p* = 0.001 for girls). Non-significant paths were indicated with dashed lines.

To test other possible consequences of the motivation in PA, a meaningful alternative model was tested (see Figure 2). We included the direct effect from students’ motivation in the context of PE to PA. This model was tested because there is previous empirical evidence suggesting direct and positive associations between autonomous types of regulation and PA intention and PA [15,17,18,20]. As in the hypothesized model, the alternative model had an adequate fit of the data: χ^2^ (650) = 1253.868, *p* < 0.001, CFI = 0.91, TLI = 0.90, RMSEA = 0.04 (95% CI (0.04, 0.05)), SRMR = 0.08. In addition, this alternative model showed the same significant relationship as the hypothesized model (see Figure 2). Specifically, students’ autonomous motivation showed a positive and significant relationship with the PA in children (*β* = 0.38; *p* < 0.001; 95% CI (0.24–0.51); for boys → *β* = 0.37; *p* < 0.001; 95% CI (0.20–0.53) and for girls → *β* = 0.39; *p* < 0.001; 95% CI (0.14–0.37)).

## 4. Discussion

The purpose of this study was to investigate the effects of teachers’ perceived autonomy support on the quality of students’ motivation in the context of PE, intention, and PA. Overall, the predictive role of perceived autonomy support on autonomous motivation, which in turn was associated with PA intention and PA, was confirmed.

The Hypothesis 1 assessed the associations between students’ perceived autonomy support and autonomous motivation. In this regard, our results showed that perceived autonomy support was significantly associated with autonomous motivation. These findings are consistent with those found by Behzadnia et al. [17], Polet et al. [21], and Leo et al. [19], where, in line with the postulates of the SDT, the autonomy support is associated with more motivational quality [12,37]. Therefore, students who perceive greater autonomy support from their teachers will also be those who have higher autonomous motivation. It is also important to note that our study also showed significant positive relationships between perceived autonomy support and controlled motivation. These results are not in line with the postulates of SDT; however, important previous studies share our findings [12,17,19], which could be explained through the different profiles of autonomy support that students may perceive. In this case, as shown by Haerens et al. [38], there may be teachers who have a high autonomy support and control profile, which in turn could cause some students to present a more autonomous or more controlled motivation according to their own interpretation, whether for reasons of enjoyment, avoiding a punishment, or receiving a reward.

Hypothesis 2 stated that autonomous motivation would be related to the intention to be physically active. In this sense, our results showed significant positive associations between autonomous motivation and intention to be physically active. These results are congruent with those found in [7,8,17,21], wherein autonomous motivation preceded intention to be physically active. This fact could be explained through the SDT construct, wherein support for the need for autonomy in physical education classes generates an adequate motivational state that influences intention and PA [13].

In line with previous findings, Hypothesis 3 proposed that the intention to be physically active will be associated with PA in children. In this regard, our results are consistent with previous research findings, wherein intention predicts PA [7,8,18]. Thus, this fact could be explained through the autonomy-supportive role of the teacher, wherein he or she generates an intention to be physically active in the school context, which eventually turns into the practice of PA in the leisure time context [18,20].

In addition, Hypothesis 4 posited that autonomous motivation would act as a mediator, on the one hand, between perceived autonomy support and intention to be physically active (H4a), and on the other hand, between perceived autonomy support and PA (H4b). In this sense, our results confirmed the mediating value of autonomous motivation on both intention and PA. These findings are consistent with those found in the research of Behzadnia et al. [17], Polet et al. [21], and Leo et al. [19]. Thus, a possible explanation can be found in an amalgam of different premises: On the one hand, the level of development of abilities and skills must be considered. Thus, the girls may be considered more competent in activities requiring balance and precision [39], which may affect their autonomous motivation. In this sense, it should be taken into account that the maturational development of the female gender occurs earlier than the male gender, which could lead in a better performance of capacities and skills that impact the central nervous system during childhood [40,41,42]. Consequently, as the girls mature earlier than the male gender, the decrease in PA during adolescence is greater. Finally, another reason may be due to tastes, sports preferences, and reasons for practicing sports. In this respect, as the girls in this study are still in their childhood, it may be that they still play sports for fun and socialization reasons [43].

To our knowledge, this is the first research to examine the validity of a theoretical model based on self-determination theory for the promotion of PA in elementary school children. So far, all studies based on the perception of autonomy support have been conducted in adolescents and university students (Vasconcellos et al. [14]). Therefore, this research shows that the perception of autonomy support by students is a key aspect for the promotion of PA in the school context. Despite these findings, the study shows some limitations: First, it should be pointed out that this was a cross-sectional study, which does not allow cause–effect relationships to be established. Therefore, future studies should carry out longitudinal studies to test the theoretical validity of the present model in children. Likewise, it is also considered a limitation to have not used the complete SDT model. Therefore, it would be desirable for future studies to use the full SDT model to evaluate the indirect effects that have not been tested in this work. In this sense, the non-inclusion of satisfaction and frustration of basic psychological needs could represent a gap in the model. However, as the study sample was children, it is very likely that such a large battery of items would not have a good response. Finally, the use of more objective data collection instruments (e.g., accelerometers and other daily activity devices) would have improved the quality of the work. In addition, they should recommend the use of objective measurement devices to provide higher quality research

## 5. Conclusions

The present study is the first to test a theoretical predictive model based on SDT for a sample of children. In this sense, it is concluded that it is important for children to perceive the teacher’s support for autonomy, which leads to the generation of an adequate motivational state that has an impact on the intention and PA. In this sense, the following practical applications can be derived from the above conclusion. Thus, a greater perception of autonomy on the part of the students will result in a more optimal motivational state that leads to greater ease in the acquisition of learning. In the same way, the PE teacher, through his or her teaching and strategies used in the classroom, can promote, both within and outside the school context, the promotion of PA and healthy habits.

## Figures and Tables

**Table 1:** Means, standards deviations, bivariate correlations, and reliability analysis of the variables.

	M	SD	α	ω	ICC	1	2	3	4	5	6
1. Perceived autonomy support	5.34	1.36	0.92	0.92	0.16	-					
2. Autonomous motivation	3.12	0.77	0.81	0.81	0.03	0.36 ***	-				
3. Controlled motivation	1.88	0.81	0.83	0.84	0.20	0.17 ***	0.35 ***	-			
4. Amotivation	1.01	1.06	0.80	0.80	0.23	−0.01	−0.06	0.56 ***	-		
5. Intention to be physically active	3.96	1.19	0.72	0.72	0.03	0.32 ***	0.49 ***	0.16 ***	−0.09 *	-	
6. Physical activity	2.31	0.49	-	-	0.02	0.17 ***	0.33 ***	0.25 ***	0.15 **	0.40 ***	-

Note. *** significance *p* < 0.001; ** significance *p* < 0.01; * significance *p* < 0.05.

## Data Availability

The datasets used and/or analysed during the current study are available from the corresponding author on reasonable request.
